# Lifetime depression and age-related changes in body composition, cardiovascular measures, grip strength and lung function

**DOI:** 10.1192/j.eurpsy.2021.1835

**Published:** 2021-08-13

**Authors:** J. Mutz, C. Lewis

**Affiliations:** Social, Genetic And Developmental Psychiatry, King’s College London, London, United Kingdom

**Keywords:** ageing, Depression, public health, physiology

## Abstract

**Introduction:**

Individuals with mental disorders, on average, die prematurely and may experience accelerated biological ageing.

**Objectives:**

We examined sex-specific associations between age and physiological measures in individuals with lifetime depression and healthy controls.

**Methods:**

UK Biobank recruited >500,000 participants, aged 37-73, between 2006–2010. Generalised additive models (GAMs) were used to examine associations between age and multiple cardiovascular, body composition, grip strength and lung function measures. Analyses were conducted separately in males and females with lifetime depression compared to healthy controls.

**Results:**

Analytical samples included up to 342,393 adults (mean age = 55.87 years, SD = 8.09; 52.61% females). We found statistically significant differences between individuals with lifetime depression and healthy controls for most physiological measures, with standardised mean differences between -0.145 and 0.156. There was some evidence that age-related changes in body composition, cardiovascular measures, lung function and heel bone mineral density followed different trajectories in individuals with lifetime depression. However, these differences did not uniformly narrow or widen with age. For example, BMI in females with lifetime depression was approximately 1.1 kg/m^2^ higher at age 40 and this difference narrowed to about 0.4 kg/m^2^ at age 70. In males, systolic blood pressure was approximately 1 mmHg lower in individuals with lifetime depression at age 45 and this difference widened to about 2.5 mmHg at age 65.

**Conclusions:**

Evidence of differences in ageing trajectories between individuals with lifetime depression and healthy controls was not uniform across physiological measures and differed by sex.

**Disclosure:**

JM receives studentship funding from the Biotechnology and Biological Sciences Research Council (BBSRC) and Eli Lilly and Company Limited. CML is a member of the Scientific Advisory Board of Myriad Neuroscience.

